# Prevalence of and risk factors for fatty liver in the general population of Northern Italy: the Bagnacavallo Study

**DOI:** 10.1186/s12876-018-0906-8

**Published:** 2018-11-28

**Authors:** Francesco Giuseppe Foschi, Giorgio Bedogni, Marco Domenicali, Pierluigi Giacomoni, Anna Chiara Dall’Aglio, Francesca Dazzani, Arianna Lanzi, Fabio Conti, Sara Savini, Gaia Saini, Mauro Bernardi, Pietro Andreone, Amalia Gastaldelli, Andrea Casadei Gardini, Claudio Tiribelli, Stefano Bellentani, Giuseppe Francesco Stefanini

**Affiliations:** 1 Department of Internal Medicine, Ospedale di Faenza, AUSL Romagna, Faenza, Italy; 2 Liver Research Center, Italian Liver Foundation, Basovizza, Trieste, Italy; 3 Department of Medical and Surgical Sciences, University of Bologna, Via Massarenti 9, 40138 Bologna, Italy; 4 Department of Internal Medicine, Ospedale di Lugo, AUSL Romagna, Locarno, Italy; 5 Research Center for the Study of Hepatitis, Department of Medical and Surgical Sciences, University of Bologna, Bologna, Italy; 6 Institute of Clinical Physiology, National Research Council, Pisa, Italy; 7 Department of Medical Oncology, Istituto Scientifico Romagnolo per lo studio e la cura dei tumori (IRST) IRCCS, Meldola, Italy; 8 Gastroenterology and Hepatology Service, Clinica Santa Chiara, Locarno, Switzerland

**Keywords:** epidemiology, cross-sectional study, prevalence, risk factors, fatty liver, chronic liver disease

## Abstract

**Background:**

The estimation of the burden of disease attributable to fatty liver requires studies performed in the general population.

**Methods:**

The Bagnacavallo Study was performed between October 2005 and March 2009. All the citizens of Bagnacavallo (Ravenna, Italy) aged 30 to 60 years as of January 2005 were eligible. Altered liver enzymes were defined as alanine transaminase > 40 U/l and/or aspartate transaminase > 37 U/l.

**Results:**

Four thousand and thirty-three (58%) out of 6920 eligible citizens agreed to participate and 3933 (98%) had complete data. 393 (10%) of the latter had altered liver enzymes and 3540 had not. After exclusion of subjects with HBV or HCV infection, liver ultrasonography was available for 93% of subjects with altered liber enzymes and 52% of those with normal liver enzymes. The prevalence of fatty liver, non-alcoholic fatty liver disease (NAFLD) and alcoholic fatty liver disease (AFLD) was 0.74 (95%CI 0.70 to 0.79) vs. 0.35 (0.33 to 0.37), 0.46 (0.41 to 0.51) vs. 0.22 (0.21 to 0.24) and 0.28 (0.24 to 0.33) vs. 0.13 (0.11 to 0.14) in citizens with than in those without altered liver enzymes. Ethanol intake was not associated and all the components of the metabolic syndrome (MS) were associated with fatty liver. All potential risk factors were associated with a lower odds of normal liver vs. NAFLD while they were unable to discriminate AFLD from NAFLD.

**Conclusions:**

Fatty liver as a whole was highly prevalent in Bagnacavallo in 2005/9 and was more common among citizens with altered liver enzymes.

**Electronic supplementary material:**

The online version of this article (10.1186/s12876-018-0906-8) contains supplementary material, which is available to authorized users.

## Background

Fatty liver (FL), the most common liver disease worldwide, is usually classified into non-alcoholic fatty liver disease (NAFLD) and alcoholic fatty liver disease (AFLD) [1, 2]. After the exclusion of hepatitis B and C and steatogenic drugs, NAFLD is currently diagnosed when FL is associated with an ethanol intake ≤20 g/day in women and ≤ 30 g/day in men [1, 2]. The NAFLD vs. AFLD dichotomization is useful in clinical practice because ethanol is unlikely to be toxic at quantities ≤30 g/day but hides the important fact that ethanol and obesity do interact to determine the burden of liver disease in the general population [2–4]. FL is commonly considered the hepatic manifestation of the metabolic syndrome (MS) but the formal testing of the hypothesis that FL is the hepatic component of MS has led to conflicting results [2, 5–7].

In the early 2000s, the Dionysos Study reported the first data on the prevalence and incidence of FL in the general population [8, 9]. In the Dionysos Nutrition & Liver Study, the citizens of Campogalliano (Modena, Emilia-Romagna, Italy) with suspected liver disease were matched with randomly chosen citizens without suspected liver disease to obtain estimates of the prevalence of and the risk factors for NAFLD and AFLD in the general population [8]. Many epidemiological studies on FL have been published since the Dionysos Nutrition & Liver Study findings were made available [10]. The worldwide prevalence of NAFLD was estimated to be 0.25 (95%CI 0.22 to 0.28) by a recent meta-analysis of 45 studies [10]. Eleven of these 45 studies were performed in Europe and yielded an estimate of 0.24 (0.16 to 0.33) for the prevalence of NAFLD. Five of these 11 studies used imaging techniques to diagnose FL and were performed in the general population [3, 8, 11–13], with one of them being a nested case-control study [3].

The so-called “ecology of medical care” model provides a strong rationale to expect that the estimates of illness made in the general population will differ from those obtained in other settings and this has indeed been repeatedly shown in practice [14, 15]. The inescapable conclusion is that the real burden attributable to a given disease cannot be estimated without epidemiological data obtained from the general population [15]. There is also mounting evidence that within a given level of the ecology of medical care [14], the individuals actually studied are often not representative of the persons making up that level, e.g. the patients enrolled in trials of NAFLD drugs are not representative of those treated in everyday practice [16].

It was with the aim of providing data on the epidemiology of FL in the general population that we performed the Bagnacavallo Study of liver disease. In detail, the aim of the Bagnacavallo Study was threefold: 1) to evaluate the prevalence of and the risk factors for FL in a cross-section of the general population of a Northern Italy town; 2) to develop a cohort of subjects from the general population where the association between FL and incident health outcomes could be studied; 3) to develop a cohort of subjects from the general population where nested case-control studies of potential risk factors for FL could be performed (taking the advantage of a purposely built serum bank) [17].

The present report deals with the first aim of the Bagnacavallo Study, i.e. the estimation of the prevalence of and the risk factors for FL in a general population. We also report the prevalence of and the risk factors for FL dichotomized into NAFLD and AFLD.

## Methods

### Study design

The Bagnacavallo Study was performed between October 2005 and March 2009. All citizens of Bagnacavallo (Ravenna, Emilia-Romagna, Italy) aged 30 to 60 years as of January 2005 were eligible and were invited by written letter to participate to the study. Public encounters were also held to promote participation to the study. Altered liver enzymes (ALE) were defined as alanine transaminase (ALT) > 40 U/l and/or aspartate transaminase (AST) > 37 U/l. These cut-points were the upper normal limits of ALT and AST applied by the laboratory that performed all the analyses of the Bagnacavallo study. The study protocol specified that all ALE+ and at least 50% of ALE- citizens had to undergo liver ultrasonography (LUS). ALE- citizens were chosen consecutively on the basis of their surname starting from a randomly chosen letter of the alphabet. The study was approved by the Ethical Committee of Area Vasta Romagna - IRST (reference number 112). All citizens gave written informed consent.

### Clinical and anthropometric assessment

All participants underwent a detailed clinical history and physical examination following the model of the Dionysos Study [8, 18]. Current alcohol intake was assessed by trained interviewers by measuring the quantity (grams) of beer, wine and liquor drunk in the week prior to the enrollment [19]. Such quantity was divided by 7 to obtain a daily estimate and converted into alcohol units with rounding to the next integer. The conversion was done using an alcohol unit corresponding to 10 g of ethanol. Weight and height were measured following international guidelines [20]. Body mass index (BMI) was calculated and classified following the NIH guidelines [21]. Waist circumference (WC) was measured at the midpoint between the last rib and the iliac crest [22].

### Laboratory assessment

Venous blood samples were obtained after 12-h fasting. The performed blood tests included: 1) glucose; 2) triglycerides; 3) total cholesterol; 4) high-density lipoprotein (HDL) cholesterol; 5) low-density lipoprotein (LDL) cholesterol; 6) ALT; 7) AST; 8) gamma-glutamyl-transferase (GGT); 9) bilirubin; 10) hepatitis B surface antigen (HBsAg); 11) antibodies against hepatitis C virus (anti-HCV).

### Metabolic syndrome

The MS was diagnosed using the harmonized international definition [23]. In detail, large WC was defined as WC ≥ 102 cm in men and ≥ 88 cm in women; high triglycerides as triglycerides ≥150 mg/dl or use of triglyceride-lowering drugs; low HDL as HDL < 40 mg/dl in men and < 50 mg/dl in women or use of HDL-increasing drugs; high blood pressure as systolic blood pressure ≥ 130 mmHg or diastolic blood pressure ≥ 85 mmHg or use or blood pressure-lowering drugs; high glucose as glucose ≥100 mg/dl or use of glucose lowering drugs; and MS as ≥3 of the above.

### Liver ultrasonography

LUS was performed by five experienced physicians (ACDA, GS, FD, AL and FC) using the same methodology of the Dionysos Nutrition & Liver Study [9, 24]. Normal liver was defined as the absence of liver steatosis or other liver abnormalities. Light FL was defined as the presence of slight “bright liver” or hepatorenal echo contrast without intrahepatic vessels blurring and no deep attenuation; moderate FL as the presence of mild “bright liver” or hepatorenal echo contrast without intrahepatic vessel blurring and with deep attenuation; and severe FL as diffusely severe “bright liver” or hepatorenal echo contrast, with intrahepatic vessels blurring (no visible borders) and deep attenuation without visibility of the diaphragm. NAFLD was defined as FL associated with ethanol intake ≤2 alcohol units (20 g) / day in women and ≤ 3 alcohol units (30 g) / day in men testing negative for HBsAg and anti-HCV and not treated with steatogenic drugs [2]. AFLD was defined as FL associated with ethanol intake ≥2 alcohol units / day in women and ≥ 3 alcohol units / day in men testing negative for HBsAg and anti-HCV and not treated with steatogenic drugs [2].

### Statistical analysis

Most continuous variables were not Gaussian-distributed and all are reported as median and interquartile range. Discrete variables are reported as the number and proportion of subjects with the characteristic of interest. Between-group comparisons of discrete variables were performed using Pearson’s Chi-square test and those of continuous variables using median regression with heteroskedasticity-robust standard errors [25].

Binary logistic regression was used to evaluate the association between FL and potential risk factors by means of six pre-specified models [26, 27]. The outcome of all the logistic regression models was FL (discrete; 0 = no; 1 = yes). Model 1 had ALE (0 = no; 1 = yes) as predictor; Model 2 added sex (0 = female, 1 = male) and age (continuous, years/10) to Model 1; Model 3 added BMI (continuous, kg/m^2^/5) and ethanol intake (continuous, alcohol units) to Model 2; Model 4 replaced BMI in Model 3 with WC (continuous, cm/10); Model 5 added MS (discrete, 0 = no; 1 = yes) and ethanol intake (continuous, alcohol units) as predictors to Model 2; Model 6 replaced MS in Model 5 with its single components, i.e. large WC (discrete, 0 = no; 1 = yes), high triglycerides (discrete, 0 = no; 1 = yes), low HDL (discrete, 0 = no; 1 = yes), high blood pressure (discrete, 0 = no; 1 = yes) and high glucose (discrete, 0 = no; 1 = yes). Before fitting the logistic regression models, we used univariable and multivariable scatterplot smoothers to get an idea of the functional form and shape of the continuous predictors and found no evidence of deviation from linearity for any predictor [28]. We also checked that the multivariable logits of the continuous predictors were linear using multivariable fractional polynomials [29]. Because alcohol intake as quantified by the present study is strictly speaking an ordinal and not a continuous variable, we tested whether its multivariable relationship with FL was linear by modeling it as both continuous and discrete in the same model [30]. We found that the relationship was linear in all models (data not shown). We also tested whether age and gender interacted in the models involving them (Models 2 to 6) and found that they did not (data not shown). Even if ethanol was not associated with FL, we nonetheless evaluated its interaction with BMI and WC because of its potential clinical significance [3]. We found no evidence of interaction of ethanol with both BMI and WC (data not shown). We evaluated the presence of collinearity among predictors in all models using the Belsley-Kuh-Welsch condition number [31]. We compared models using Akaike information criterion (AIC) and the Bayesian information criterion (BIC) and additionally calculated Nagelkerke pseudo-R^2^ and the area the under the receiver-operating characteristic curve (-ROC-AUC) [26]. We used Model 3 to calculate and plot the sex-specific marginal probabilities of FL corresponding to the 5th, 25th, 50th, 75th and 95th internal percentiles of age and BMI at the median intake of ethanol [27, 32].

Multinomial logistic regression was used to evaluate the association between FL type and potential predictors by means of six pre-specified models [26, 27]. The outcome of all the multinomial logistic regression models was FL type (discrete; 0 = NAFLD; 1 = normal liver; 2 = AFLD). The prediction models were the same described above under binary logistic regression with the exception that ethanol was not entered into any model. We set NAFLD as the reference category in order to obtain estimates of effect sizes for the normal liver vs. NAFLD and the AFLD vs. AFLD comparisons. We performed the same tests of model assumptions described above under binary logistic regression. We compared models using Akaike information criterion (AIC) and the Bayesian information criterion (BIC) and additionally calculated Nagelkerke pseudo-R^2^. ROC-AUC were calculated for the two binary logistic models underlying the multinomial logistic model. Statistical analysis was performed using Stata 15.1 (Stata Corporation, College Station, TX, USA).

## Results

### Flow of the citizens through the study

**Fig. 1:**
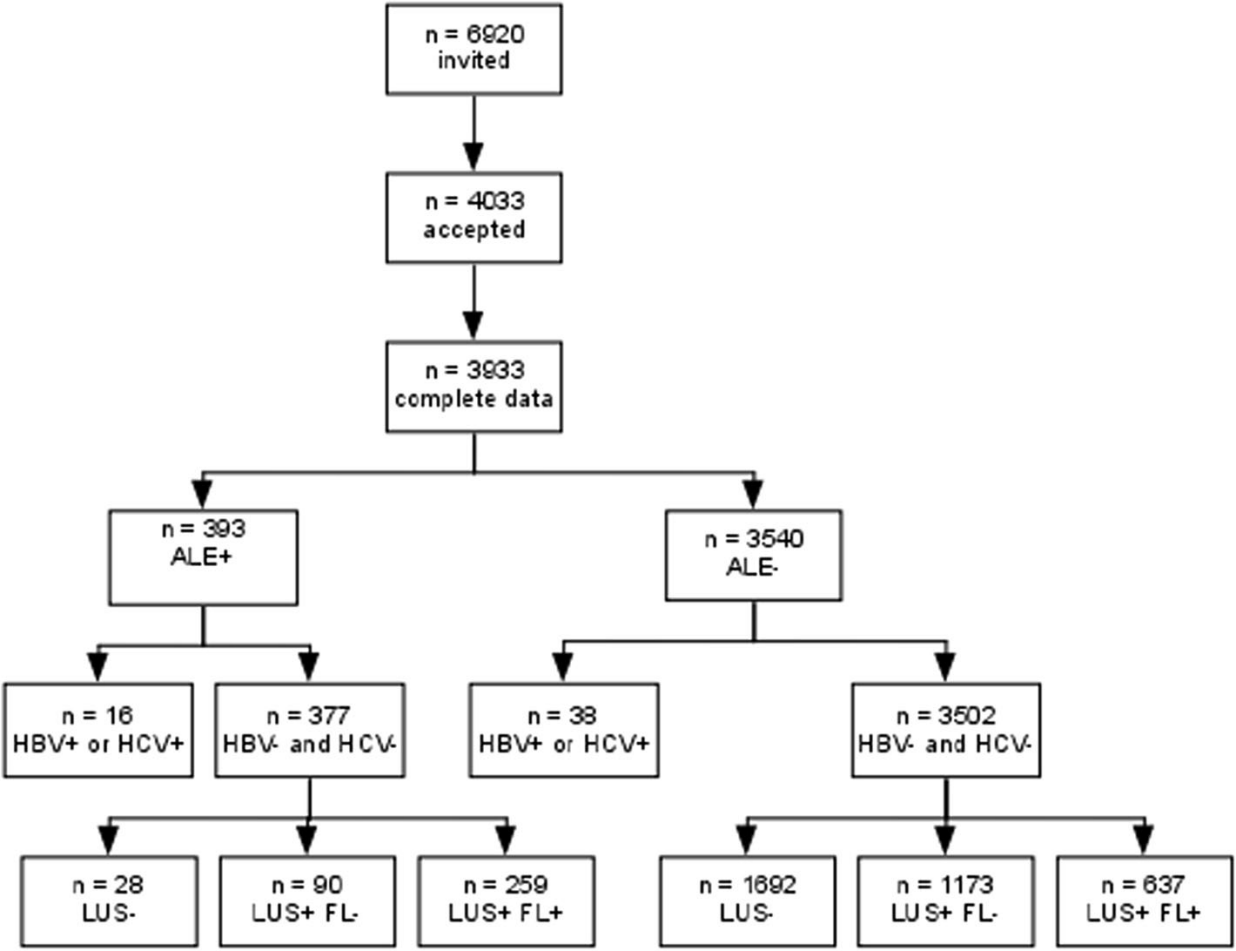
Flow of the subjects through the study. Abbreviations: ALE = altered liver enzymes; HBV = Hepatitis B virus; HCV = hepatitis C virus; LUS = liver ultrasonography; FL = fatty liver

The flow of the citizens through the study is depicted in Fig. 1. Four thousand and thirty-three (58%) of the 6920 citizens aged 30 to 60 years who resided in Bagnacavallo as of January 2005 agreed to participate to the study and 3933 (98%) of them had all the data required for analysis. The citizens were consecutively studied during the first three days of every week (except for holidays) between October 2005 and March 2009. The study protocol required that all ALE+ and at least 50% of ALE- citizens were recalled to undergo LUS.

### Comparison of the subjects with and without liver ultrasonography among the citizens with altered liver enzymes

Three hundred ninety-three (10%) of the 3933 citizens with complete data were ALE+. Sixteen (4.1%) of them had HBV or HCV infection and will not be considered here. Of the remaining 377 ALE+ citizens, 349 (93%) underwent LUS. Additional file 1: Table S1 compares the 349 ALE+ citizens with LUS to the 28 ALE+ citizens without LUS. As compared to ALE+ citizens without LUS, ALE+ citizens with LUS had higher values of triglycerides, ALT and GGT (*p* < 0.05). Besides not being of great interest in itself [33], the lack of statistical significance should be taken with an additional degree of caution here owing to the low number of ALE+ subjects without LUS (*n* = 28).

### Comparison of the subjects with and without liver ultrasonography among the citizens with normal liver enzymes

Among the 3540 (90%) ALE- citizens, 38 had HBV or HCV infection (1.1%) and will not be considered here. Of the remaining 3502 ALE- citizens, 1810 (52%) underwent LUS. Additional file 2: Table S2 compares the 1810 ALE- citizens with LUS to the 1692 ALE- citizens without LUS. As compared to ALE- citizens without LUS, ALE- citizens with LUS were more likely to be male and had higher values of age, weight, BMI, WC, glucose, triglycerides, cholesterol, LDL-cholesterol, systolic blood pressure, ALT, GGT and bilirubin (*p* ≤ 0.05). Besides not being of great interest in itself [33], the presence of statistical significance should be taken with an additional degree of caution here owing to the high number of ALE- subjects with (*n* = 1810) and without LUS (*n* = 1692).

### Comparison of the citizens with and without altered liver enzymes among those with liver ultrasonography

Table 1 compares the ALE+ and ALE- citizens with availability of LUS. ALE+ citizens were more frequently male and slightly younger than ALE- citizens. ALE+ citizens had greater values of BMI, WC, glucose, triglycerides, total cholesterol, LDL-cholesterol, systolic blood pressure and diastolic blood pressure and lower values of HDL-cholesterol.

**Table 1:** Comparison of citizens with and without altered liver enzymes

	ALE-	ALE+	*p*-value^*^
	*n* = 1810	*n* = 349	
Age (years)	49 (41–56)	47 (40–55)	0.03
Male sex	812 (44.9%)	267 (76.5%)	< 0.001
Weight (kg)	72.0 (61.0–82.0)	84.0 (74.0–95.0)	< 0.001
Height (m)	1.68 (1.60–1.74)	1.73 (1.67–1.79)	< 0.001
BMI (kg/m^2^)	25.1 (22.6–28.1)	27.9 (25.4–30.9)	< 0.001
BMI class (NIH)			< 0.001
Underweight	19 (1.0%)	0 (0.0%)	
Normal	871 (48.1%)	66 (18.9%)	
Overweight	607 (33.5%)	170 (48.7%)	
Obesity class 1	230 (12.7%)	81 (23.2%)	
Obesity class 2	65 (3.6%)	28 (8.0%)	
Obesity class 3	18 (1.0%)	4 (1.1%)	
Fatty liver	637 (35.2%)	259 (74.2%)	< 0.001
Fatty liver degree			< 0.001
Light	428 (67.2%)	107 (41.3%)	
Moderate	151 (23.7%)	102 (39.4%)	
Severe	58 (9.1%)	50 (19.3%)	
Fatty liver type			< 0.001
None	1173 (64.8%)	90 (25.8%)	
NAFLD	407 (22.5%)	160 (45.8%)	
AFLD	230 (12.7%)	99 (28.4%)	
Waist circumference (cm)	100.0 (93.0–107.0)	105.0 (100.0–113.0)	< 0.001
Large waist circumference	1236 (68.3%)	259 (74.2%)	0.028
Glucose (mg/dl)	89 (83–96)	93 (87–102)	< 0.001
High fasting glucose	307 (17.0%)	109 (31.2%)	< 0.001
Triglycerides (mg/dl)	97 (68–139)	138 (98–206)	< 0.001
High triglycerides	405 (22.4%)	159 (45.6%)	< 0.001
Total cholesterol (mg/dl)	207 (184–234)	215 (192–240)	0.005
HDL cholesterol (mg/dl)	61 (51–73)	50 (44–61)	< 0.001
Low HDL	219 (12.1%)	64 (18.3%)	0.002
LDL cholesterol (mg/dl)	126 (104–150)	138 (117–159)	< 0.001
Systolic blood pressure (mm Hg)	125 (120–140)	130 (120–140)	< 0.001
Diastolic blood pressure (mm Hg)	80 (80–90)	85 (80–90)	< 0.001
High blood pressure	1053 (58.2%)	270 (77.4%)	< 0.001
Metabolic syndrome	444 (24.5%)	171 (49.0%)	< 0.001
Metabolic syndrome score			< 0.001
0	216 (11.9%)	19 (5.4%)	
1	595 (32.9%)	59 (16.9%)	
2	555 (30.7%)	100 (28.7%)	
3	294 (16.2%)	97 (27.8%)	
4	117 (6.5%)	59 (16.9%)	
5	33 (1.8%)	15 (4.3%)	
ALT (U/l)	20 (15–26)	50 (44–63)	< 0.001
AST (U/l)	20 (18–24)	33 (29–41)	< 0.001
GGT (U/l)	17 (12–26)	42 (27–69)	< 0.001
Total bilirubin (mg/dl)	0.60 (0.40–0.81)	0.62 (0.49–0.90)	0.003
Alcohol intake (alcohol units/day)	2 (0–4)	3 (1–5)	< 0.001
Wine intake (alcohol units/day)	2 (0–2)	2 (1–3)	< 0.001
Beer intake (alcohol units/day)	0 (0–1)	0 (0–1)	0.52
Liquor intake (alcohol units/day)	0 (0–0)	0 (0–1)	< 0.001

Values are given as median (interquartile range) for continuous variables and number (proportion) for dichotomous variables

*Abbreviations: ALE* altered liver enzymes, *BMI* body mass index, *NAFLD* non-alcoholic fatty liver disease, *AFLD* alcoholic fatty liver disease, *NIH* National Institutes of Health, *HDL* high-density lipoprotein, *LDL* low-density lipoprotein, *ALT* alanine transaminase, *AST* aspartate transaminase, *GGT* gamma-glutamyl transferase

*Median regression for continuous variables and Pearson’s Chi-square test for binary categorical variables

### Prevalence and risk factors for fatty liver

The prevalence of FL was 0.74 (95%CI 0.70 to 0.79) among ALE+ and 0.35 (0.33 to 0.37) among ALE- citizens (*p <* 0.001). The severity of FL (light vs. moderate vs. severe) was also higher among ALE+ than ALE- citizens. All the components of the MS and the MS itself were more prevalent among ALE+ than ALE- subjects. Lastly, alcohol intake was higher in ALE+ than in ALE- subjects.

Table 2 reports the logistic regression models used to investigate the association between FL and potential risk factors.

**Table 2:** Logistic regression models used to investigate the association between fatty liver and potential risk factors

	M1	M2	M3	M4	M5	M6
ALE	5.3** [4.1 to 6.9]	5.1** [3.9 to 6.7]	3.9** [2.9 to 5.2]	4.0** [3.0 to 5.4]	4.2** [3.2 to 5.6]	3.7** [2.8 to 5.0]
Male	–	2.1** [1.7 to 2.5]	2.0** [1.6 to 2.5]	2.1** [1.7 to 2.6]	2.0** [1.6 to 2.4]	2.4** [1.9 to 3.0]
Age (years) / 10	–	1.8** [1.6 to 2.0]	1.6** [1.4 to 1.8]	1.5** [1.4 to 1.7]	1.5** [1.4 to 1.7]	1.4** [1.3 to 1.6]
BMI (kg/m^2^) / 5	–	–	3.9** [3.3 to 4.5]	–	–	–
Alcohol intake (units)	–	–	1.0 [0.9 to 1.0]	1.0 [1.0 to 1.1]	–	–
Waist circumference (cm) / 10	–	–	–	2.5** [2.3 to 2.8]	–	–
Metabolic syndrome	–	–	–	–	5.1** [4.1 to 6.3]	–
Large waist circumference	–	–	–	–	–	2.9** [2.3 to 3.8]
High triglycerides	–	–	–	–	–	3.1** [2.4 to 3.9]
Low HDL	–	–	–	–	–	1.6* [1.2 to 2.2]
High blood pressure	–	–	–	–	–	1.9** [1.5 to 2.3]
High glucose	–	–	–	–	–	2.0** [1.5 to 2.6]
*n*	2159	2159	2159	2159	2159	2159
AIC	2750	2595	2131	2244	2376	2266
BIC	2762	2618	2165	2278	2405	2317
ROC-AUC	0.61	0.72	0.83	0.81	0.79	0.81
Pseudo-R^2^ (Nagelkerke)	0.11	0.20	0.42	0.37	0.31	0.36

Values are odds ratios and 95% confidence intervals (logistic regression)

*Abbreviations: M#* model number, *ALE* altered liver enzymes, *BMI* body mass index, *HDL* high-density lipoprotein, *AIC* Akaike information criterion, *BIC* Bayesian information criterion, *ROC-AUC* area under the ROC curve, *pseudo-R*^*2*^ pseudo-squared R

*p < 0.01; **p < 0.001

Model 1 shows that the odds of FL was 5.3 (95%CI 4.1 to 6.9) times higher in ALE+ than in ALE- citizens. The corresponding probabilities of FL estimated from the logistic regression model are 74% (95%CI 70 to 79%) for ALE+ and 35% (95%CI 33 to 37%) for ALE- citizens.

Model 2 evaluates whether sex and age are associated with FL independently from ALE. While both sex (OR = 2.1, 95%CI 1.7 to 2.5 for males) and age (OR = 1.8, 95%CI 1.6 to 2.0 per decade) show an independent effect on FL, the OR of ALE changed only slightly (4%) compared to Model 1.

Model 3, obtained by adding BMI and alcohol intake as predictors to Model 2, shows that BMI is associated with FL (OR = 3.9, 95%CI 3.3 to 4.5 per 5 kg/m^2^) with modest changes of the OR of sex (5%) and age (11%) and with a moderate change of the OR of ALE (23%). Importantly, this model shows that ethanol intake is not associated with FL (OR = 1.0, 95%CI 1.0 to 1.1 per alcohol unit). All the employed metrics of model fit identified Model 3 as the best of all models (lowest AIC, lowest BIC, highest ROC-AUC and highest pseudo-R^2^).

Model 4, obtained by replacing BMI in Model 3 with WC, shows that WC is associated with FL (OR = 2.5, 95%CI 2.3 to 2.8) independently of ALE, sex and alcohol intake. The effect sizes of ALE, sex and age are similar to those of Model 3 using BMI as predictor and ethanol intake is again not associated with FL. (We did not evaluate BMI and WC together in Model 4 because of the evidence of collinearity as determined by a Belsley-Kuh-Welsch condition number of 31).

Model 5 evaluates the association of MS with FL taking into account ALE, sex and age. Having MS is associated with an odds of FL equal to 5.1 (95%CI 4.1 to 6.3). (Neither BMI nor WC were entered into this model because WC is already included in the definition of MS and BMI and WC were collinear as explained above.)

Model 6 evaluates the independent contribution of each component of the MS (large WC, high triglycerides, low HDL, high blood pressure and high glucose) to FL. Not only was each component of the MS associated with FL, but all the measures of model fit were better for Model 6 than for Model 5 (lower AIC, lower BIC, higher ROC-AUC and higher pseudo-R^2^), showing that there is some advantage in considering the single components of the MS instead of the MS as its association with FL is concerned.

**Fig. 2:**
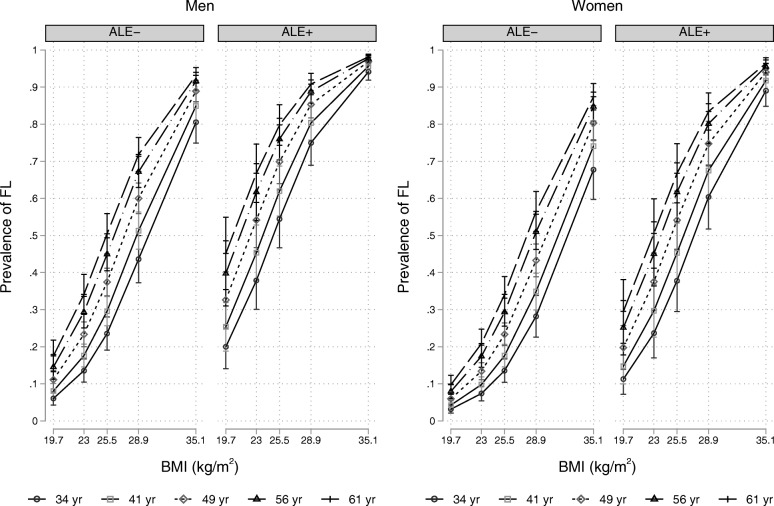
Prevalence of fatty liver in men and women with and without altered liver enzymes as estimated by Model 3 of Table 2. Values are proportions and pointwise 95% confidence intervals. Abbreviations: ALE = altered liver enzymes; FL = fatty liver; BMI = body mass index. The values of age correspond to the 5th (34 yr), 25th (41 yr), 50th (49 yr), 75th (56 yr) and 95th (61 yr) internal percentiles. The values of BMI correspond to the 5th (19.7 kg/m^2^), 25th (23.0 kg/m^2^), 50th (25.5 kg/m^2^), 75th (28.9 kg/m^2^) and 95th (35.1 kg/m^2^) internal percentiles and alcohol intake is set to the median value

Figure 2 plots the prevalence of FL in men and women with and without ALE as estimated by Model 3 with ethanol intake set at the median value [32]. In both sexes, the prevalence of FL increases with age and BMI.

### Prevalence of and risk factors for NAFLD and AFLD

The prevalence of NAFLD and AFLD in ALE+ and ALE- subjects was 0.46 (0.41 to 0.51) vs. 0.22 (95%CI 0.21 to 0.24) and 0.28 (0.24 to 0.33) vs. 0.13 (CI 0.11 to 0.14).

Table 3 reports the multinomial logistic regression models used to investigate the association between FL type and potential risk factors.

**Table 3:** Multinomial logistic regression models used to investigate the association between fatty liver type and potential risk factors

	M1	M2	M3	M4	M5	M6
Normal liver vs. NAFLD						
Altered liver enzymes	0.20*** [0.15,0.26]	0.19*** [0.14,0.25]	0.25*** [0.18,0.34]	0.24*** [0.17,0.33]	0.23*** [0.17,0.31]	0.26*** [0.19,0.35]
Male sex	–	0.61*** [0.49,0.75]	0.65*** [0.51,0.82]	0.58*** [0.46,0.73]	0.64*** [0.51,0.81]	0.49*** [0.37,0.64]
Age (years) / 10	–	0.53*** [0.46,0.60]	0.60*** [0.52,0.69]	0.61*** [0.53,0.70]	0.61*** [0.53,0.70]	0.65*** [0.56,0.75]
BMI (kg/m^2^) / 5	–	–	0.26*** [0.22,0.30]	–	–	–
WC (cm) / 10	–	–	–	0.39*** [0.34,0.43]	–	–
Metabolic syndrome	–	–	–	–	0.20*** [0.16,0.26]	–
High waist circumference	–	–	–	–	–	0.29*** [0.21,0.39]
High triglycerides	–	–	–	–	–	0.38*** [0.29,0.50]
Low HDL	–	–	–	–	–	0.54*** [0.39,0.76]
High blood pressure	–	–	–	–	–	0.56*** [0.43,0.72]
High fasting glucose	–	–	–	–	–	0.52*** [0.39,0.69]
*n*	1830	1830	1830	1830	1830	1830
ROC-AUC	0.61	0.72	0.83	0.82	0.78	0.81
AFLD vs. NAFLD						
Altered liver enzymes	1.09 [0.81,1.48]	0.89 [0.65,1.21]	0.87 [0.64,1.20]	0.91 [0.66,1.24]	0.87 [0.63,1.19]	0.87 [0.63,1.20]
Male sex	–	1.95*** [1.44,2.63]	1.96*** [1.44,2.65]	1.89*** [1.39,2.56]	1.95*** [1.44,2.64]	1.52* [1.09,2.13]
Age (years) / 10	–	0.82* [0.70,0.97]	0.83* [0.70,0.97]	0.84* [0.71,0.99]	0.82* [0.69,0.97]	0.80* [0.67,0.96]
BMI (kg/m^2^) / 5	–	–	1.01 [0.87,1.17]	–	–	–
WC (cm) / 10	–	–	–	0.92 [0.81,1.03]	–	–
Metabolic syndrome	–	–	–	–	1.05 [0.79,1.39]	–
High waist circumference	–	–	–	–	–	0.68* [0.47,0.97]
High triglycerides	–	–	–	–	–	1.58** [1.17,2.13]
Low HDL	–	–	–	–	–	0.64* [0.44,0.93]
High blood pressure	–	–	–	–	–	1.10 [0.78,1.56]
High fasting glucose	–	–	–	–	–	1.12 [0.82,1.53]
*n*	896	896	896	896	896	896
ROC-AUC	0.51	0.60	0.60	0.61	0.61	0.63
Whole model						
*N*	2159	2159	2159	2159	2159	2159
AIC	3932	3754	3290	3402	3537	3419
BIC	3955	3800	3347	3459	3594	3521
Pseudo-R^2^ (Nagelkerke)	0.10	0.19	0.38	0.34	0.28	0.34

Values are odds ratios and 95% confidence intervals (multinomial logistic regression). ROC-AUC were calculated for the two binary logistic models underlying the multinomial logistic model

*Abbreviations: M#* model number, *NAFLD* non-alcoholic fatty liver disease, *BMI* body mass index, *WC* waist circumference, *HDL* high-density lipoprotein, *AFLD* alcoholic fatty liver disease, *AIC* Akaike information criterion, *BIC* Bayesian information criterion, *ROC-AUC* area under the ROC curve, *pseudo-R*^*2*^ pseudo-squared R

^*^p < 0.05; ^**^p < 0.01; ^***^p < 0.001

Not unexpectedly, all potential risk factors were associated with a lower odds of normal liver vs. NAFLD. All odds ratios were in fact < 1 for all predictors (Models 1–6). More interestingly, the same predictors were unable to discriminate AFLD from NAFLD as made clear by the unsatisfactory ROC-curves of the binary ALFD vs. NAFLD model.

In detail, ALE (Models 1–6), BMI (Model 3), WC (Model 4) and MS (Model 5) were not associated with the odds of having AFLD vs. NAFLD. Although male gender and lower age were associated with a greater odds of AFLD vs. NAFLD in all models (Models 1–6), their 95%CI are wide. It is of some interest that high WC and low HDL made AFLD less likely than NAFLD and that high triglycerides made AFLD more likely than NAFLD (Model 6) but the 95%CI of these estimates are again wide.

## Discussion

Although much more epidemiological data are presently available on FL as compared to when the Dionysos Study was performed [3, 8], there are still few studies performed in representative samples of the general population [10]. FL has a different course in the general population than in primary, secondary and tertiary care centers, where most of the presently available studies on FL were performed [34]. This is in line with the so-called “ecology of medical care” model, according to which only a minority of citizens with a given illness will actually search for and get medical care [14]. Thus, the real burden attributable to FL cannot be estimated without epidemiological data obtained from the general population [15].

The strengths of the present study are that it was performed in a representative sample of the general population, that it enrolled a high number of subjects, and that it built a serum bank that we plan to use in future studies. The most important limitation of the study is the suboptimal response rate (58%). Although this response rate is the same of the Dionysos Nutrition & Liver Study [8] and is higher than that reported by most studies [35], it is possible that the citizens who refused to participate to the Bagnacavallo Study differed systematically from those who accepted to participate with an ensuing selection bias. Another limitation of the present study is the use of LUS to diagnose FL. Although LUS is virtually the only feasible option to diagnose FL in population studies, it is known to offer an accurate assessment of FL only starting from an intrahepatic triglyceride content of 10% [5, 36]. Thus, lesser degrees of FL may have gone undetected in the present study and our estimates of FL prevalence may be conservative.

In the present study, 74% of ALE+ citizens had FL compared to 35% of ALE- citizens. In the Dionysos Nutrition & Liver Study, performed in the same region of Northern Italy during 2002/3, 44% of citizens with suspected liver disease had FL as compared to 35% of those without suspected liver disease [8]. The estimates made by the Bagnacavallo Study and by the Dionysos Nutrition & Liver Study are unfortunately not comparable because of the different operational definitions of ALE and suspected liver disease. The criteria for suspected liver disease adopted by the Dionysos Nutrition & Liver Study did in fact include an altered GGT (> 35 U/l), did not consider AST, and did consider an ALT > 30 U/l as altered [8]. The Bagnacavallo Study confirms nonetheless, as firstly shown by the Dionysos Nutrition & Liver Study in a general population [8], that FL is quite common (35%) among subjects with normal liver enzymes.

The analysis of the potential risk factors for FL yielded two very interesting findings. The first finding is that ethanol intake was not an independent predictor of FL in the general population. The Dionysos Nutrition & Liver Study reported the same finding even if a direct comparison of the Dionysos Nutrition & Liver Study and the Bagnacavallo Study is not possible because of the different instruments used to measure alcohol intake [9]. The second finding is that all the components of the MS were associated to FL independently of ALE, gender, age and alcohol intake. The dichotomization implicit in the concept of MS has been criticized by research methodologists on the basis of both clinical and statistical grounds [5, 37]. The findings of the present study offer a further empirical argument for preferring the use of the single components of the MS instead of the whole MS for the study of the association of FL as a whole with cardiometabolic risk factors.

### Prevalence of and risks factors for non-alcoholic fatty liver disease and alcoholic fatty liver disease

Although the present study focused on FL as a whole considering ethanol intake as a potential predictor, we performed an analysis of the prevalence of and the risk factors for NAFLD to allow a comparison with the available studies [10].

In the present study, the prevalence of NAFLD was 46% among ALE+ and 22% among ALE- citizens. The corresponding figures for citizens with and without suspected liver disease in the Dionysos Nutrition & Liver Study were 25 and 20% [8]. The Bagnacavallo Study and Dionysos Nutrition & Liver Study estimates are unfortunately not comparable not only because of the different operational definitions of ALE and suspected liver disease as discussed above, but also because the Dionysos Nutrition & Liver Study employed a different cut-point of alcohol intake to diagnose NAFLD in men (≤ 20 g/day) and used a different instrument (7-day prospective diary) to measure ethanol intake [8]. We have, indeed, previously shown that small errors in the estimation of ethanol intake may lead to a substantial difference in the estimated prevalence of NAFLD vs. AFLD [5, 8].

The analysis of the potential risk factors for NAFLD yielded a very interesting finding. All potential risk factors were associated with a lower odds of normal liver vs. NAFLD in all models, which were able to satisfactorily discriminate NAFLD from normal liver. However, the same models were not able to discriminate AFLD from NAFLD. There are several, not mutually exclusive, explanations for this finding. First, the dichotomization of ethanol intake, central to the separation of NAFLD from AFLD [38], could have introduced substantial bias into the inference [37]. Second, we have not cross-validated the recall method used to assess ethanol intake in the present study against an accepted reference method, e.g. the 7-day prospective diary used the Dionysos Nutrition & Liver Study [8]. Thus, we ignore the measurement error of the method used to assess ethanol intake [39]. This is unfortunately the rule in the literature on FL and is especially troublesome because the separation of NAFLD from AFLD is based entirely on the dichotomization of alcohol intake [38]. Third, it is possible that the separation between NAFLD and AFLD is not relevant at the population level because most of the risk factors for FL as whole are not associated with its separation into NAFLD and AFLD. However, without accurate measurements of ethanol intake, this hypothesis remains highly speculative. In view of the fact that ethanol is a well-known hepatotoxic agent, our data should not be taken as evidence that ethanol intake is not an individual risk factor for fatty liver but simply that with conventional instruments used to detect fatty liver and measure ethanol intake, this relationship was not evident at the population level in Bagnacavallo in 2005/2009.

## Conclusions

In conclusion, FL was highly prevalent in a Northern Italy town in 2005/9 and was more common among ALE individuals. It had no association with alcohol intake but was strongly associated with anthropometry and all the MS components. NAFLD was more common than AFLD but, while anthropometry and all the MS components were able to discriminate normal liver from NAFLD, they did not discriminate AFLD from NAFLD. The cross-sectional data presented in this paper will inform the ongoing and future analyses of the Bagnacavallo cohort, which we hope will offer new and relevant information on the burden of FL in the general population.

## Additional files

Additional file 1: **Table S1.** Comparison of the citizens with and without liver ultrasonography among those with altered liver enzymes. In this table we compared the 349 citizens with altered liver enzymes (ALE+) and with liver ultrasonography (LUS) to the 28 ALE+ citizens without LUS. (DOCX 18 kb)

Additional file 2: **Table S2.** Comparison of the citizens with and without liver ultrasonography among those with normal liver enzymes. In this table we compared the 1810 citizens without altered liver enzymes (ALE-) and with liver ultrasonography (LUS) to the 1692 ALE- citizens without LUS. (DOCX 18 kb)

## Abbreviations

AFLD: Alcoholic fatty liver disease
AIC: Akaike information criterion
ALE: Altered liver enzymes
ALT: Alanine transaminase
anti-HCV: Antibodies against hepatitis C virus
AST: Aspartate transaminase
BIC: Bayesian information criterion
BMI: Body mass index
FL: Fatty liver
GGT: Gamma-glutamyl-transferase
HBsAg: Hepatitis B surface antigen
HDL: High-density lipoprotein
LDL: Low-density lipoprotein
LUS: Liver ultrasonography
MS: Metabolic syndrome
NAFLD: Non-alcoholic fatty liver disease
ROC-AUC: Area the under the receiver-operating characteristic curve
WC: Waist circumference
