# Supplementary material for: Prevalence of and risk factors for fatty liver in the general population of Northern Italy: the Bagnacavallo Study
Source: BMC Gastroenterol. 2018 Nov 28;18:177. doi: 10.1186/s12876-018-0906-8 (PMC6262973; doi:10.1186/s12876-018-0906-8)
Supplement: Supplementary file 1 — Table S1. Comparison of the citizens with and without liver ultrasonography among those with altered liver enzymes. In this table we compared the 349 citizens with altered liver enzymes (ALE+) and with liver ultrasonography (LUS) to the 28 ALE+ citizens without LUS. (DOCX 18 kb) [file 12876_2018_906_MOESM1_ESM.docx]

**Additional file 1: Table S1 - Comparison of the citizens with and without liver ultrasonography among those with altered liver enzymes.**

|  | ALE+  LUS not available  *n* = 28 | ALE+  LUS available  *n* = 349 | *p*-value^*^ |
| --- | --- | --- | --- |
| Age (years) | 45 (40-52) | 47 (40-55) | 0.41 |
| Male sex | 25 (89.3%) | 267 (76.5%) | 0.12 |
| Weight (kg) | 83.0 (75.5-95.0) | 84.0 (74.0-95.0) | 1.00 |
| Height (m) | 1.73 (1.70-1.78) | 1.73 (1.67-1.79) | 1.00 |
| BMI (kg/m^2^) | 27.4 (24.2-29.6) | 27.9 (25.4-30.9) | 0.79 |
| Waist circumference (cm) | 104.0 (97.5-111.5) | 105.0 (100.0-113.0) | 0.63 |
| Glucose (mg/dl) | 93 (89-99) | 93 (87-102) | 1.00 |
| Triglycerides (mg/dl) | 103 (77-166) | 138 (98-206) | 0.03 |
| Total cholesterol (mg/dl) | 216 (182-228) | 215 (192-240) | 1.00 |
| HDL cholesterol (mg/dl) | 53 (46-60) | 50 (44-61) | 0.21 |
| LDL cholesterol (mg/dl) | 125 (106-152) | 138 (117-159) | 0.41 |
| Systolic blood pressure (mm Hg) | 130 (122-140) | 130 (120-140) | 1.00 |
| Diastolic blood pressure (mm Hg) | 82 (80-90) | 85 (80-90) | 1.00 |
| ALT (U/l) | 44 (41-58) | 50 (44-63) | 0.04 |
| AST (U/l) | 32 (27-38) | 33 (29-41) | 0.66 |
| GGT (U/l) | 57 (30-74) | 42 (27-69) | 0.02 |
| Total bilirubin (mg/dl) | 0.60 (0.46-0.79) | 0.62 (0.49-0.90) | 0.72 |
| Alcohol intake (units/day) | 4 (2-5) | 3 (1-5) | 1.00 |

^*^Median regression for continuous variables and Pearson’s Chi-square test for dichotomous variables

Values are given as median (interquartile range) for continuous variables and number (proportion) for binary variables.

Abbreviations: ALE = altered liver enzymes; LUS = liver ultrasonography; BMI = body mass index; HDL = high-density lipoprotein; LDL = low-density lipoprotein; ALT = alanine transaminase; AST = aspartate transaminase; GGT = gamma-glutamyl transferase.
