# Supplementary material for: Prevalence of and risk factors for fatty liver in the general population of Northern Italy: the Bagnacavallo Study
Source: BMC Gastroenterol. 2018 Nov 28;18:177. doi: 10.1186/s12876-018-0906-8 (PMC6262973; doi:10.1186/s12876-018-0906-8)
Supplement: Supplementary file 2 — Table S2. Comparison of the citizens with and without liver ultrasonography among those with normal liver enzymes. In this table we compared the 1810 citizens without altered liver enzymes (ALE-) and with liver ultrasonography (LUS) to the 1692 ALE- citizens without LUS. (DOCX 18 kb) [file 12876_2018_906_MOESM2_ESM.docx]

**Additional file 2: Table S2 - Comparison of the citizens with and without liver ultrasonography among those with normal liver enzymes.**

|  | ALE-  LUS not available  *n* = 1692 | ALE-  LUS available  *n* = 1810 | *p*-value^*^ |
| --- | --- | --- | --- |
| Age (years) | 47 (40-55) | 49 (41-56) | <0.001 |
| Male sex | 701 (41.4%) | 812 (44.9%) | 0.04 |
| Weight (kg) | 69.0 (60.0-79.0) | 72.0 (61.0-82.0) | <0.001 |
| Height (m) | 1.68 (1.60-1.74) | 1.68 (1.60-1.74) | 1.00 |
| BMI (kg/m^2^) | 24.4 (22.1-27.0) | 25.1 (22.6-28.1) | <0.001 |
| Waist circumference (cm) | 99.0 (92.0-104.0) | 100.0 (93.0-107.0) | 0.005 |
| Glucose (mg/dl) | 87 (82-94) | 89 (83-96) | <0.001 |
| Triglycerides (mg/dl) | 89 (65-129) | 97 (68-139) | <0.001 |
| Total cholesterol (mg/dl) | 203 (179-226) | 207 (184-234) | <0.001 |
| HDL cholesterol (mg/dl) | 62 (52-74) | 61 (51-73) | 0.15 |
| LDL cholesterol (mg/dl) | 122 (101-144) | 126 (104-150) | 0.002 |
| Systolic blood pressure (mm Hg) | 120 (120-130) | 125 (120-140) | <0.001 |
| Diastolic blood pressure (mm Hg) | 80 (80-90) | 80 (80-90) | 1.00 |
| ALT (U/l) | 19 (15-24) | 20 (15-26) | 0.004 |
| AST (U/l) | 20 (17-23) | 20 (18-24) | 1.000 |
| GGT (U/l) | 15 (11-22) | 17 (12-26) | <0.001 |
| Total bilirubin (mg/dl) | 0.54 (0.40-0.70) | 0.60 (0.40-0.81) | <0.001 |
| Alcohol intake (units/day) | 2 (0-4) | 2 (0-4) | 1.000 |

*Median regression for continuous variables and Pearson’s Chi-square test for dichotomous variables

Values are given as median (interquartile range) for continuous variables and number (proportion) for binary variables.

Abbreviations: ALE = altered liver enzymes; LUS = liver ultrasonography; BMI = body mass index; HDL = high-density lipoprotein; LDL = low-density lipoprotein; ALT = alanine transaminase; AST = aspartate transaminase; GGT = gamma-glutamyl transferase.
